# Furmonertinib plus pemetrexed in the treatment of *EGFR* exon 19 deletion lung adenocarcinoma: two case reports

**DOI:** 10.3389/fonc.2026.1709552

**Published:** 2026-01-29

**Authors:** Yuan Zhang, Duofang Wang, Huaxiu Ma, Xiaojun Wang

**Affiliations:** 1 The First School of Clinical Medical, Gansu University of Chinese Medicine, Lanzhou, Gansu, China; 2 Department of Respiratory and Critical Care Medicine, Gansu Provincial Hospital, Lanzhou, Gansu, China

**Keywords:** EGFR exon 19 deletion, furmonertinib, lung adenocarcinoma, pemetrexed, treatment response

## Abstract

Epidermal growth factor receptor (*EGFR*) exon 19 deletion (Ex19del) is one of the most prevalent sensitizing mutations in non-small cell lung cancer (NSCLC), particularly in Asian populations. However, management after progression or suboptimal response is unclear. We describe two patients with advanced lung adenocarcinoma harboring *EGFR* Ex19del who received furmonertinib plus pemetrexed. Case 1 achieved partial response (PR) with substantial tumor shrinkage and a marked decline in carcinoembryonic antigen (CEA) after six cycles; disease remained stable over 19 months of follow-up. Case 2 had suboptimal benefit from first-line osimertinib but attained PR with resolution of pleural effusion after switching to the combination; subsequent computed tomography (CT) confirmed stable disease (SD). Both patients tolerated treatment without severe treatment-related adverse events. These observations suggest that furmonertinib plus pemetrexed may have antitumor activity and acceptable tolerability in *EGFR* Ex19del lung adenocarcinoma, and may inform personalized approaches following resistance to first-line therapy in *EGFR*-sensitizing NSCLC.

## Introduction

Non-small-cell lung cancer (NSCLC) is a common malignancy worldwide, with adenocarcinoma as the predominant subtype (1, 2). Epidermal growth factor receptor (*EGFR*) mutations are major therapeutic targets, and exon 19 deletions (Ex19del) account for about half of all *EGFR* mutations, representing the most frequent sensitizing variant (3). In recent years, the use of third-generation EGFR tyrosine kinase inhibitor (EGFR-TKI) has significantly improved the prognosis of patients with *EGFR*-mutant NSCLC. Osimertinib, a representative drug, is established as the standard first-line treatment and significantly prolongs survival (4). Meanwhile, the Chinese-developed third-generation EGFR-TKI, furmonertinib, with its unique trifluoroethoxy pyridine structure, demonstrates superior blood-brain barrier penetration and favorable safety and tolerability in clinical use (5). However, clinical evidence for combining furmonertinib with chemotherapy remains limited. Here, we report two cases of advanced lung adenocarcinoma with *EGFR* Ex19del treated with furmonertinib plus pemetrexed to explore the potential activity, feasibility, and clinical context for this regimen.

## Case report

### Case 1

**Figure 1:**
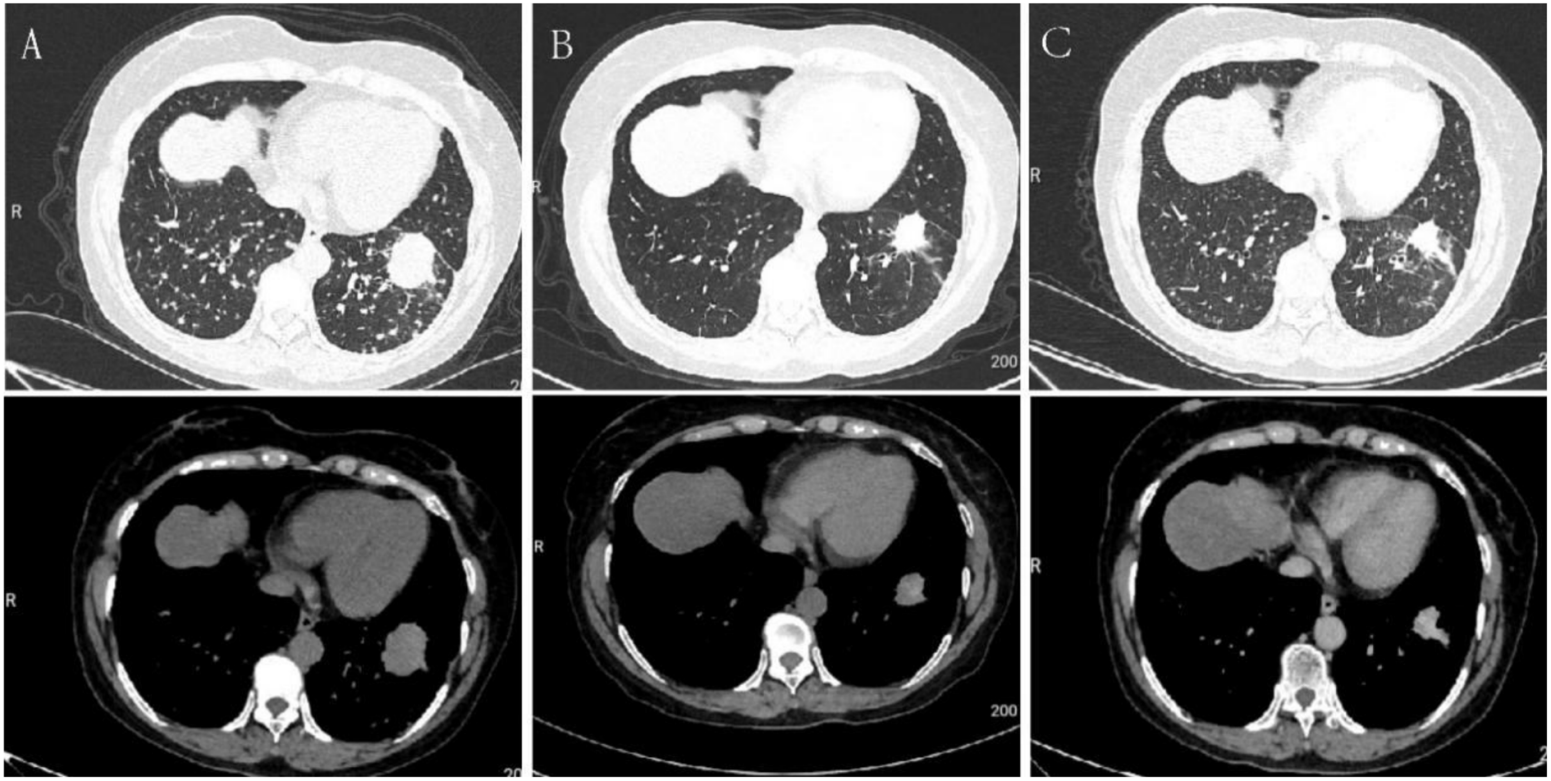
Serial chest CT scans of Case 1 during diagnosis and treatment. **(A)** Baseline chest CT (December 22, 2023) showing a mass-like lesion in the left lower lobe. **(B)** After 2 months of treatment (February 2024), the lesion was reduced to 2.8 × 2.5 cm. **(C)** After 4 months of treatment (April 2024), further reduction of the lesion to 2.7 × 2.2 cm was observed.

A 66-year-old woman presented with a two-week history of cough and sputum production. Chest computed tomography (CT) on December 22, 2023, revealed a mass measuring 3.6 × 3.2 cm in the anteromedial basal segment of the left lower lobe, with multiple bilateral pulmonary metastases (**Figure 1A**). Cranial magnetic resonance imaging (MRI) showed a lesion with abnormal signal intensity in the right parietal bone. Positron emission tomography–CT (PET-CT) confirmed the primary lesion in the left lower lobe with lymph node and bone metastases. Serum tumor markers demonstrated elevated carcinoembryonic antigen (CEA, 1424.53 ng/mL) and cytokeratin-19 fragment (CYFRA21-1, 4.91 ng/mL). Neuron-specific enolase (NSE, 2.45 ng/mL) and squamous cell carcinoma antigen (SCC, 0.40 ng/mL) were within the normal range. A CT-guided percutaneous lung biopsy of the left lower lobe confirmed invasive mucinous adenocarcinoma. Immunohistochemistry showed Syn (–), Napsin-A(+), TTF-1(+), CK7(+), p63(–), CAM5.2(+), Ki-67 (25–50%), and CDX-2(–). Amplification Refractory Mutation System Polymerase Chain Reaction(ARMS-PCR) detected an Ex19del mutation, supporting the use of targeted therapy. No additional concurrent genetic alterations, including *TP53*, *KRAS*, *STK11*, or other common driver mutations, were identified. The diagnosis was primary lung adenocarcinoma of the left lower lobe, stage IV (T4N3M1c).

Following evaluation of the clinical status and exclusion of contraindications, the patient initiated furmonertinib (80 mg once daily) plus pemetrexed on December 30, 2023. Given the high tumor burden with extensive pulmonary and brain metastases, combination therapy was initiated at treatment onset. Pemetrexed was selected based on its proven efficacy in non-squamous NSCLC and its frequent use in patients with brain metastases in clinical practice, and this regimen was supported by multidisciplinary team (MDT) consensus to achieve early systemic disease control. Six 21-day cycles were administered. Premedication included folic acid and dexamethasone to mitigate treatment-related toxicity. Follow-up chest CT in February 2024 showed a reduction of the primary lesion to 2.8 × 2.5 cm (**Figure 1B**). In April 2024, the lesion had further decreased to 2.7 × 2.2 cm (**Figure 1C**). Cranial MRI demonstrated shrinkage of the parietal lesion compared with December 2023, and serum CEA decreased to 210.16 ng/mL. According to RECIST v1.1,the best overall response was assessed as partial response (PR). The patient subsequently continued maintenance treatment with furmonertinib. During the 19-month follow-up through June 2025, the disease remained stable with preserved performance status and no grade ≥3 adverse events. In July 2025, the patient declined further therapy due to financial constraints and passed away in September 2025. Disease progression was suspected based on symptom deterioration reported by the family.

### Case 2

**Figure 2:**
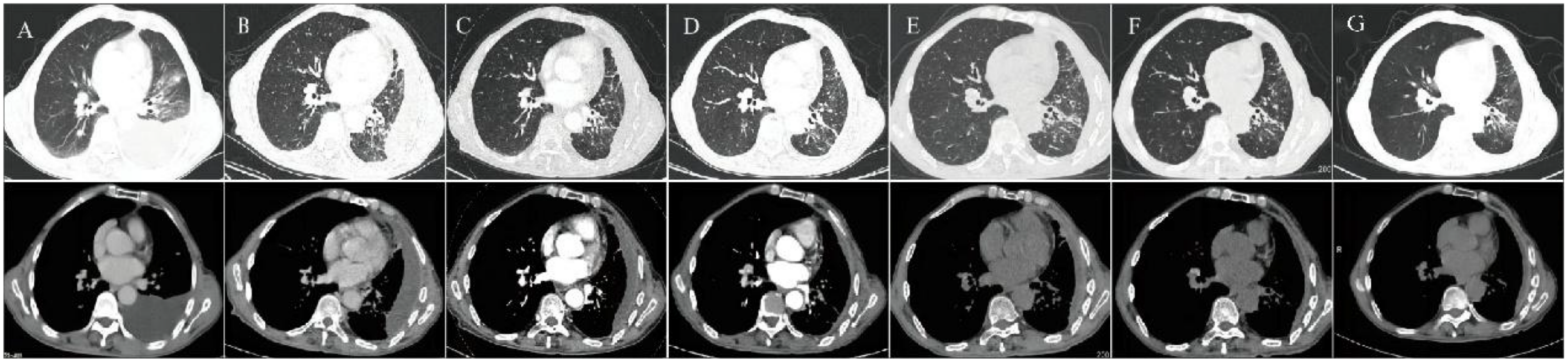
Serial chest CT scans of Case 2 during diagnosis and treatment. **(A)** Baseline chest CT prior to targeted therapy. **(B)** After 3 months of osimertinib (January 31, 2024). **(C)** After 4 cycles of furmonertinib plus pemetrexed (May 10, 2024). **(D)** Month 9 of furmonertinib treatment (October 9, 2024). **(E)** Month 12 of furmonertinib treatment (February 27, 2025). **(F)** Month 16 of furmonertinib treatment (June 17, 2025). **(G)** Month 21 of furmonertinib treatment (November 20, 2025).

A 69-year-old man presented with a five-month history of intermittent cough with sputum, chest tightness, and shortness of breath. His medical history included thyroid cancer and left thyroidectomy. Chest CT at admission revealed left pleural effusion with incomplete expansion of the left lower lobe and multiple small pulmonary nodules bilaterally (**Figure 2A**). PET-CT showed mildly increased uptake at the T10–T11 vertebrae, considered degenerative. Serum tumor markers showed increased CEA (181.93 ng/mL), CYFRA21-1 (>100 ng/mL) and SCC (2.00 ng/mL), while NSE (4.36 ng/mL) remained within the normal range. A thoracoscopic biopsy of the left lower lobe confirmed diffuse mucinous adenocarcinoma. Immunohistochemistry showed CK7(+), Napsin-A(+), TTF-1(+), CK5/6(–), p40(–), CgA(–), Syn(–), CD56(–), Ber-EP4(+), CR(–), MC (partially+), TG(–), and Ki-67 (10%). ARMS-PCR identified an *EGFR* Ex19del mutation. No additional concurrent genetic alterations, including *TP53*, *KRAS*, *STK11*, or other common driver mutations, were identified. The diagnosis was primary lung adenocarcinoma of the left lower lobe, stage IVA (cT4N0M1a).

The patient initially received osimertinib (80 mg once daily) for three months as first-line targeted therapy; however, pleural effusion was inadequately controlled (**Figure 2B**). Treatment was then adjusted to furmonertinib (80 mg once daily) plus pemetrexed every 21 days for four cycles starting February 23, 2024. Chest ultrasound on March 15, 2024, revealed a 10.7 cm fluid layer in the left pleural cavity. A repeat ultrasound in April 2024 demonstrated marked improvement, with the depth of pleural effusion reduced to 4.3 cm. The treatment response was assessed as partial response (PR). Subsequent follow-up CT scans were performed in May 2024, October 2024, February 2025, June 2025, and November 2025 (**Figures 2C–G**). The overall response to furmonertinib plus pemetrexed was assessed as stable disease (SD), indicating durable disease control. Treatment was well tolerated, and no grade ≥3 treatment-related adverse events were observed. The patient remains under active follow-up.

## Discussion

In recent years, the treatment paradigm for advanced *EGFR*-mutant NSCLC has evolved, with first-line therapy shifting from single-agent TKI to more effective combination regimens. Current research centers on third-generation EGFR-TKI-based combinations with chemotherapy, anti-angiogenic agents, and novel bispecific antibodies (6–9). In this context, we report two patients with advanced lung adenocarcinoma who achieved significant responses to furmonertinib plus pemetrexed.

Resistance to first-line osimertinib can be broadly categorized into EGFR-dependent and EGFR-independent mechanisms (10). EGFR-dependent resistance typically involves acquired on-target alterations in the *EGFR* gene, most commonly tertiary mutations such as C797S (10). This mutation causes conformational changes in the kinase domain that block the irreversible covalent binding of osimertinib to the C797 site, which reduces its inhibitory effect and allows reactivation of downstream EGFR signaling (11). In contrast, EGFR-independent resistance typically arises from activation of bypass signaling pathways or downstream reprogramming, including *MET* or *HER2* amplification and alterations in the RAS/RAF/PI3K pathways (10). It can also involve phenotypic or histologic transformation linked to *TP53* and *RB1* co-loss (12). Besides *MET* amplification, growing evidence suggests *HER3* acts as a key mediator of EGFR-TKI resistance (13). As a critical signaling integrator, *HER3* maintains activation of the PI3K/AKT and MAPK pathways through heterodimerization with *EGFR*, *MET*, or *HER2*, which promotes tumor cell survival and adaptive resistance (13). Importantly, when clinical response to osimertinib is suboptimal, comprehensive molecular profiling using next-generation sequencing (NGS) is critical for identifying the underlying resistance mechanisms and guiding subsequent therapeutic strategies (14, 15).

When osimertinib shows limited efficacy or resistance emerges, switching to a third-generation EGFR-TKI with a different chemical structure is biologically plausible (16, 17). Furmonertinib, a Chinese-developed third-generation EGFR-TKI, contains a trifluoroethoxypyridine group that alters its spatial conformation, lipophilicity, and EGFR binding compared with osimertinib (18, 19). Furmonertinib also metabolizes *in vivo* to AST5902, an active metabolite that establishes a dual inhibition mechanism, potentially enhancing and prolonging EGFR pathway suppression. The phase III FURLONG study demonstrated that first-line furmonertinib treatment in patients with *EGFR*-mutant NSCLC achieved an objective response rate (ORR) of 89% and a median progression-free survival (PFS) of 20.8 months, with a favorable safety profile (5). Crucially, real-world evidence substantiates furmonertinib efficacy following third-generation EGFR-TKI failure, validating sequential application in this refractory population (17, 20, 21).

While trials such as OPTIMAL established EGFR-TKI monotherapy as the first-line standard (22), patients with high tumor burden or intracranial metastases often require rapid disease control. In such settings, combining a third-generation EGFR-TKI with pemetrexed achieves concurrent suppression of both the dominant EGFR pathway and heterogeneous tumor subpopulations, thereby enhancing response depth and durability (19, 23). The FLAURA2 trial and several meta-analyses have shown that combining targeted therapy with chemotherapy (including pemetrexed-based regimens) improves PFS, overall survival (OS), and ORR compared to TKI monotherapy (24, 25). In this study, we chose furmonertinib plus pemetrexed over platinum-based chemotherapy to optimize both early efficacy and long-term tolerability. Case 1 presented with high tumor burden, intracranial metastases, and markedly elevated CEA. We deployed this regimen first-line to achieve rapid, deep remission. In Case 2, after first-line osimertinib proved inadequate, we switched to this regimen to regain control of systemic and pleural disease. These two cases illustrate an adaptive treatment approach based on tumor burden and treatment response.

Third-generation EGFR-TKIs have significantly improved initial treatment responses and survival outcomes; however, residual drug-tolerant persister (DTP) cells are increasingly recognized as a critical reservoir underlying disease relapse and acquired resistance (26, 27). Research increasingly emphasizes eradicating residual disease and alleviating the immunosuppressive microenvironment induced by EGFR-TKI therapy. First, studies demonstrate that this therapy upregulates immunoregulatory molecules, notably cluster of differentiation 24 (CD24) and CD73, which promote tumor cell survival by evading innate immune clearance (28, 29). CD24 functions as an innate immune checkpoint by engaging macrophage Siglec-10 to suppress phagocytosis, and blockade of this axis restores macrophage-mediated antitumor activity (30). Liang et al. further showed that CD24 upregulation is driven by phosphorylation of Yin Yang-1 (YY1) at S247, and that combining CD24 blockade with third-generation EGFR-TKIs synergistically enhances antitumor immunity against residual disease (28). Separately, CD73 promotes an immunosuppressive microenvironment via adenosine signaling, and its co-inhibition with PD-L1 enhances antitumor T cell responses in EGFR-mutant NSCLC models (29). These findings highlight the value of overcoming EGFR-TKI-induced immune tolerance. Second, amivantamab is a bispecific antibody that simultaneously targets EGFR and MET. It inhibits EGFR-MET crosstalk and bypass signaling driven by MET, and further augments immunity through Fc effector functions (31, 32). The MARIPOSA study showed that amivantamab plus lazertinib significantly prolonged PFS versus osimertinib in patients with common *EGFR* mutations, with updated analyses suggesting an overall survival signal (9, 33). The CHRYSALIS-2 trial demonstrated activity of amivantamab-based combinations after progression on osimertinib, establishing its role in the post-third-generation EGFR-TKI setting (34). Furthermore, amivantamab-based regimens are incorporated into the NCCN NSCLC guidelines, supported by phase III evidence for amivantamab plus chemotherapy in *EGFR* exon 20 insertion NSCLC (33). These results underscore the dual role of amivantamab in overcoming MET-mediated resistance and enhancing therapeutic efficacy. Third, antibody drug conjugates (ADCs) targeting trophoblast cell surface antigen 2 (TROP2) demonstrate antitumor activity in advanced NSCLC (35). Phase III trials (TROPION-Lung01 and EVOKE-01) showed clinical benefit versus docetaxel but no significant OS improvement; subgroup heterogeneity was noted in TROPION-Lung01 (36, 37). Given that EGFR-TKI-induced DTP cells can adopt quiescent or slow-cycling phenotypes, ADC-mediated cytotoxicity may be less effective against residual disease (38). In preclinical models of *EGFR*-mutant NSCLC, Baldacci et al. showed that CAR-T cell therapy targeting TROP2 effectively eliminated osimertinib-induced DTP and produced durable responses (26). In addition, emerging studies are mapping molecular determinants of EGFR-TKI sensitivity and drug-tolerant persistence. Representative examples include resistance programs involving AXL, NOTCH signaling that sustains persisters tolerant to osimertinib, and Aurora B–dependent apoptotic regulation that can prevent or overcome resistance to EGFR inhibition (39–41). Overall, emerging therapeutic strategies for advanced EGFR-mutant NSCLC increasingly favor combination regimens, aiming to eradicate residual disease and delay relapse.

This study has several limitations. First, it is based on two case reports with a small sample size, limiting the generalizability of the findings. Second, absence of molecular profiling at progression precludes detailed resistance analysis. Nevertheless, these observations provide practical insights into upfront intensification with a third-generation EGFR-TKI plus pemetrexed. For lung adenocarcinoma patients with *EGFR* exon 19 deletions with high tumor burden, brain metastases, or inadequate response to osimertinib, furmonertinib plus pemetrexed may confer meaningful survival benefits with manageable toxicity. These preliminary findings require validation in larger prospective cohorts with longitudinal molecular monitoring.

## Data Availability

The original contributions presented in the study are included in the article/supplementary material. Further inquiries can be directed to the corresponding author.
